# Identification of a myometrial molecular profile for dystocic labor

**DOI:** 10.1186/1471-2393-11-74

**Published:** 2011-10-16

**Authors:** Donal J Brennan, Sharon F McGee, Elton Rexhepaj, Darran P O'Connor, Michael Robson, Colm O'Herlihy

**Affiliations:** 1 National Maternity Hospital, Dublin , Ireland; 2 UCD School of Biomolecular and Biomedical Science, UCD Conway Institute, Dublin , Ireland; 3 UCD School of Medicine and Medical Science, Dublin, Ireland

## Abstract

**Background:**

The most common indication for cesarean section (CS) in nulliparous women is dystocia secondary to ineffective myometrial contractility. The aim of this study was to identify a molecular profile in myometrium associated with dystocic labor.

**Methods:**

Myometrial biopsies were obtained from the upper incisional margins of nulliparous women undergoing lower segment CS for dystocia (n = 4) and control women undergoing CS in the second stage who had demonstrated efficient uterine action during the first stage of labor (n = 4). All patients were in spontaneous (non-induced) labor and had received intrapartum oxytocin to accelerate labor. RNA was extracted from biopsies and hybridized to Affymetrix HuGene U133A Plus 2 microarrays. Internal validation was performed using quantitative SYBR Green Real-Time PCR.

**Results:**

Seventy genes were differentially expressed between the two groups. 58 genes were down-regulated in the dystocia group. Gene ontology analysis revealed 12 of the 58 down-regulated genes were involved in the immune response. These included (ERAP2, (8.67 fold change (FC)) HLA-DQB1 (7.88 FC) CD28 (2.60 FC), LILRA3 (2.87 FC) and TGFBR3 (2.1 FC)) Hierarchical clustering demonstrated a difference in global gene expression patterns between the samples from dystocic and non-dystocic labours. RT-PCR validation was performed on 4 genes ERAP2, CD28, LILRA3 and TGFBR3

**Conclusion:**

These findings suggest an underlying molecular basis for dystocia in nulliparous women in spontaneous labor. Differentially expressed genes suggest an important role for the immune response in dystocic labor and may provide important indicators for new diagnostic assays and potential intrapartum therapeutic targets.

## Background

As cesarean section (CS) rates continue to rise throughout the developed world, an improved understanding of the molecular mechanisms underlying parturition at term is urgently required. Between 1974 and 2008 overall cesarean rates increased from 5% to 19.1% in the National Maternity Hospital (NMH), Dublin and one of the major contributors to this was a 4-fold increase in cesarean deliveries amongst *term singleton cephalic nulliparas *(TSCN) [1]. CS rates vary between institutions, although TSCN CS rates correlate with institutional CS rates and we have previously documented that 98% of inter-institutional variation in overall CS rates can be attributed to TSCN rates [2], which demonstrates that TSCN as a cohort has a significant impact on cesarean rates within any obstetric population. However, despite much attention within the obstetric literature addressing the timing and mode of twin deliveries [3], vaginal breech delivery [4] and the optimum management of pre-term, growth-restricted fetuses [5], little emphasis has been placed on the management of TSCN, an important group of parturients, which has been relatively neglected hitherto by research initiatives [2].

The most common primary indication for CS is dystocia or slow labor, most commonly secondary to inefficient myometrial contractility. Dystocia is a common obstetric problem, affecting 3-8% of deliveries [6], and is a particular complication of first pregnancies. In approximately 40% of first labors, dystocia can be adequately and safely corrected by intrapartum administration of oxytocin, resulting in vaginal delivery [7]. In 10-20% of cases of dystocia, however, myometrial response to oxytocin is poor and CS becomes the only safe option following prolonged labor [8]. It is believed this complication is likely to increase mainly due to delayed childbearing and increased prevalence of obesity in the obstetric population both factors which adversely affect intrapartum myometrial contractility [9,10]. For every 1% increase in the incidence of nulliparous CS for dystocia, overall caesarean rates will inevitably increase by at least 0.5% due to the consequent increase in CS in women with scarred uteri. An improved understanding of the molecular mechanisms underlying dystocic labor and may result in alternative adjuvants to oxytocin.

To date, we are not aware of studies using gene expression profiling to evaluate the physiology and mechanisms underlying dystocia in nulliparous women in spontaneous labor. In a large Swedish population-based study, which examined over two million deliveries, Algovik *et al *demonstrated a large genetic influence on dystocia [6]. This study estimated that heritability contributed 28% in the liability of developing primary dystocia, with significant concordance evident amongst monozygotic twins and an increased risk of dystocia in women who had a mother or sister with a history of dystocic labors. Additionally, transgenic knockout mouse models examining prostaglandin F2α receptor [11] and testosterone 5-α-reductase type 1 [12], respectively, exhibit an absence of parturition, although screening for mutations in these candidate dystocia-related genes in humans proved unsuccessful [13]. The genetic basis of dystocia is likely to be more complex that a single gene mutation. Considering the clinical impact of dystocia, a single gene mutation would be subject to purifying selection and would be selected against in the evolutionary process. Therefore genome-wide screening should provide more useful insights. The aim of this study was to use gene expression microarrays to examine the temporal and spatial changes in myometrial gene expression during normally-progressing and dystocic labors to improve our understanding of the molecular mechanisms underlying term dystocic labor.

## Methods

**Figure 1:**
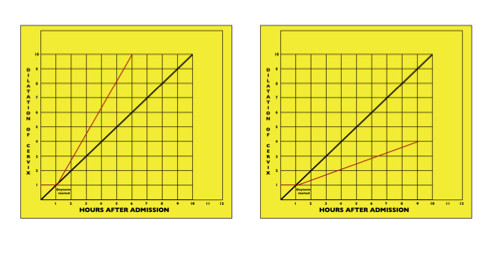
**Dystocia and Efficient Uterine Action**. Partograms representing dystocia and efficient uterine action.

The National Maternity Hospital is a tertiary referral university institution that publishes an Annual Clinical Report, which includes detailed analysis of obstetric outcomes and modes of delivery of all delivered mothers (> 9, 000 annually) and on all perinatal deaths (stillbirths and first-week neonatal deaths of infants who weighed > 500 g, corrected for the exclusion of lethal malformations). Management of nulliparous labor has been standardized in the institution throughout the last four decades according to an Active Management of Labor protocol [8], the principles of which include accurate early diagnosis of labor, early correction of inefficient uterine action, and one to one care by a personal nurse throughout labor [14]. Inefficient uterine action is corrected by the administration of intravenous oxytocin in a concentration of 10 IU/L, when subsequent cervical dilatation does not progress at a rate of at least 1 cm per hour. Oxytocin acceleration is commenced in a dosage of 5 mU/min, and is increased to a maximum of 30 mU/min and titrated against the frequency of uterine contractions, which are restricted to 7 contractions in 15 minutes. Progress in all labors is recorded on an individual partogram, which plots cervical dilatation as a function of time, up to a maximum of 12 hours (Figure 1). Using this protocol, less than 3% of women admitted to the NMH remain undelivered after 12 hours, when they are delivered by CS unless delivery is imminent.

Definitions used for this study included: *nulliparous*: para 0, irrespective of gravidity; *term*: greater than or equal to 37 completed weeks gestation and *singleton gestation*, no evidence of a multiple gestation after the first trimester. Representative partograms for both groups used in this study are shown in Figure 1.

### Sample Collection

Patients provided informed consent to partake in this study and ethical approval was obtained from ethics committee in the National Maternity Hospital, Holles Street, Ireland. Myometrial biopsies were obtained from the midpoint of the upper incisional margins of the uteri of women undergoing lower segment CS for dystocia (n = 4) and in control women undergoing CS in the second stage, who had demonstrated efficient (≥ 1 cm dilatation per hour) uterine action (EUA) during the first stage of labor (n = 4). Care was taken to remove decidual tissue. All patients in this group underwent CS for persistent occiput posterior position. A scissors was used to obtain myometrial samples, all of which measured approximately 1 cm^3^. All patients presented to the labor ward in spontaneous labor with intact membranes and subsequently underwent an amniotomy and intrapartum oxytocin acceleration. All amniotomies were performed at a cervical dilatation of 3 cm or less. Patient characteristics are outlined in table 1; all were Caucasian with singleton pregnancies, aged less than 35 years. No women developed pyrexia during labor or received prophylactic antibiotics.

**Table 1:** Patient characteristics

Group	Age	BMI	Parity	Gestation	Duration of Labor	Epidural	Cervical Dilation at CS	Baby Weight
Dystocia	29	22.8	0	40^+3^	10hrs	Yes	2 cm	3810 gm
Dystocia	26	24.4	0	41^+0^	11hrs	Yes	4 cm	4260 gm
Dystocia	33	22.7	0	41^+1^	9hrs	Yes	2 cm	2890 gm
Dystocia	27	31.2	0	41^+1^	6hrs	Yes	3 cm	3710 gm
EUA	25	23.4	0	40^+6^	10hrs	Yes	10 cm	3145 gm
EUA	31	24.3	0	40^+5^	6hrs	Yes	10 cm	3440 gm
EUA	32	28.7	0	39^+3^	8hrs	Yes	10 cm	3930 gm
EUA	30	31.0	0	41^+5^	8hrs30min	Yes	10 cm	4840 gm

Abbreviations: EUA - efficient uterine action, BMI - body mass index (at booking).

Myometrial samples were transferred directly in the operating room into RNALater (Qiagen, Crawley, West Sussex, UK) and stored at 4°C for 24 hours. RNALater was then removed and samples were stored at -80°C. Total RNA extraction and purification from tissue samples were carried out using TRIzol reagent (Invitrogen, Carlsbad, CA) as per the manufacturer's protocol. Preparation of labeled complementary RNA and hybridization to HGU133 Plus 2.0 GeneChips (Affymetrix, Santa Clara, CA) was performed as per the manufacturer's recommended protocol.

### Data Analysis

Raw data (cel files) were analyzed using Bioconductor 1.9 http://bioconductor.org running on R 2.6.0 [15]. Normalization was performed using the Affymetrix package's Robust Multichip Average (RMA) default method [16]. Differential gene expression was measured using a modified t test, and genes demonstrating greater than 2 fold difference and a p value < 0.05 were considered to be differentially expressed. Hierarchical clustering and principal components analysis were performed using MatLab 7 (MathWorks, Apple Hill Drive, MA). Raw data are available in Geo http://www.ncbi.nlm.nih.gov/projects/geo/, accession number GSE32178.

### Quantitative SYBR Green Real-Time PCR

Total RNA was isolated from tissues using Trizol (Invitrogen) and reverse transcribed using SuperScript II™ Reverse Transcriptase (Invitrogen) according to the manufacturer's instructions. ERAP2 (FW-TGGATGGGACCAACTCATTACA, RV- TGCACCAACTAGCTGAAACAC), CD28 (FW- GGCATCCCTTCACAAAGGACT, RV- CCCCGTTTTTGAGTAAACCTGA), LILRA3 (FW- CCAGTGTGTTTCTGATGTCAGC, RV- CCGGATGCACCGAGATGAAG), HLA-DQB1 (FW- AGGGTGAATGTTTCCCCCTC, RV- CTGCCTGGGTAGAAATCCGT) primers were designed using Primer Express software (Applied Biosystems Version 2.0) and used to amplify specific DNA fragments with SYBR Green PCR Master Mix (Applied Biosystems) using a 7900HT Fast Real-Time PCR System (Applied Biosystems). Relative expression levels were calculated using the qBase real-time PCR relative quantification software [17] with all samples normalized to GAPDH expression. Negative controls included a no template control and a no reverse transcriptase control. All qRT-PCR reactions were performed in triplicate.

## Results

Genome-wide transcriptomic analysis was performed on myometrial biopsies taken from the lower uterine segment in Caucasian women undergoing CS for dystocia (n = 4) and in women who demonstrated efficient uterine action in the first stage of labor, but underwent a second stage CS for persistent occiput posterior position. All of the patients included in the study were nulliparas in spontaneous labor between 37 and 42 weeks. There were no differences between the two groups regarding maternal age, BMI, or gestation at delivery (Table 1). The mean birthweight in the dystocic group was 3667 gm compared to 3839 gm in the efficient uterine action group; all patients received epidural anaesthesia.

**Figure 2:**
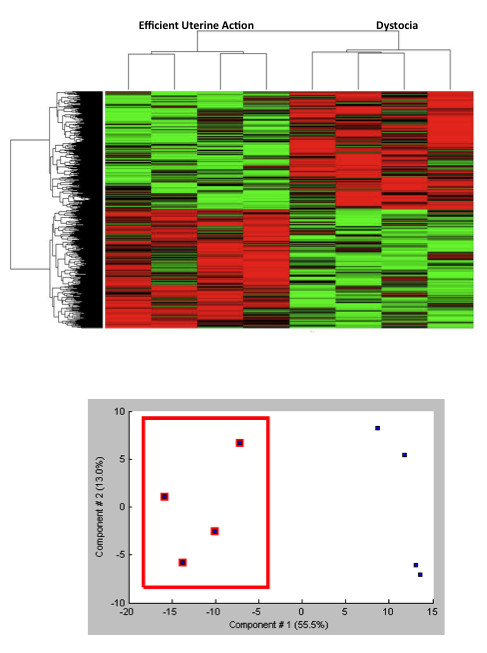
**Molecular Profile of Dytocia**. Hierarchical clustering demonstrating a distinct dystocia transcriptomic profile (a). Principal components analysis demonstrating aggregation of dystocia specimens.

Unsupervised hierarchical clustering and principal components analysis (PCA) were performed to gain a global view of gene expression in dystocia and efficient myometrium. Probes were filtered based on gene entropy and genes displaying entropy values below the 10^th ^centile were removed prior to hierarchical clustering and PCA. As demonstrated in Figure 2, hierarchical clustering of genes with a high entropy value produced a very distinctive separation between dystocia and EUA. This was confirmed using PCA whereby the four dystocic samples clustered together (Figure 2b), demonstrating that dystocia has a distinctive myometrial molecular profile compared to normally progressing labor.

**Figure 3:**
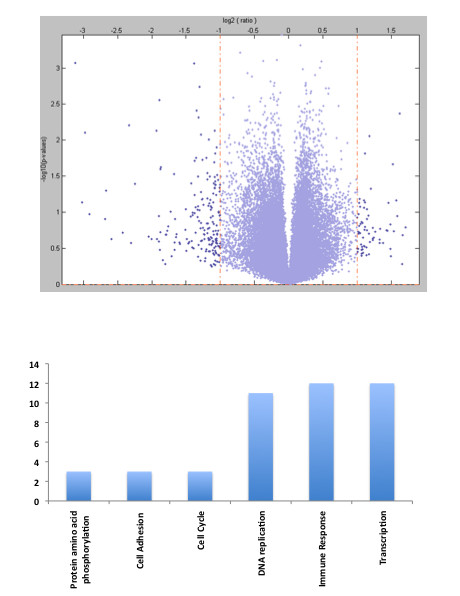
**Differentially Expressed Genes**. Volcano plot demonstrating differentially expressed genes (a). Gene ontology analysis of differentially expressed genes (b)

To examine the molecular profile of dystocia in more detail, both dystocic and non-dystocic groups were compared to identify differentially expressed genes. Given the relatively small number of samples in both groups, strict thresholds were applied and only genes that exhibited a 2-fold change and a *p value *< 0.05 were considered differentially expressed (Figure 3a). Using this threshold, 70 genes were differentially expressed in women with dystocia compared to efficient uterine action (Table 2). Within the 70 genes, 58 were down-regulated and 12 were up-regulated in the dystocic specimens compared to normally progressing labor. Gene ontology analysis revealed that the most common processes in the differentially expressed genes were immune response, transcription and DNA replication (Figure 3b). In particular a number of key regulators of the cell cycle were down-regulated in the dystocic samples such as cyclin E, CENPK and ID2, all of which promote progression through the cell cycle.

**Table 2:** Differentially expressed genes in dystocic labor

Gene Symbol	Gene Title	Fold Change	p value
ERAP2	endoplasmic reticulum aminopeptidase 2	-8.67	0.0009
C15orf48	chromosome 15 open reading frame 48	-8.11	0.0732
HLA-DQB1	major histocompatibility complex, class II, DQ beta 1	-7.88	0.0079
LOC389831	hypothetical gene supported by AL713796	-6.35	0.0503
HBG1	hemoglobin, gamma A	-5.05	0.0062
LOC389831	hypothetical gene supported by AL713796	-4.75	0.0406
CCNE2	cyclin E2	-3.71	0.0028
KIAA0101	KIAA0101	-3.66	0.0253
ID2	inhibitor of DNA binding 2, dominant negative helix-loop-helix protein	-3.08	0.0043
C7	complement component 7	-2.87	0.0217
LILRA3	leukocyte immunoglobulin-like receptor, subfamily A (without TM domain), member 3	-2.84	0.0916
TRIM13	tripartite motif-containing 13	-2.74	0.0744
MLF1IP	MLF1 interacting protein	-2.68	0.0400
DTL	denticleless homolog (Drosophila)	-2.60	0.0009
CD28	CD28 molecule	-2.60	0.0576
LOC283788	FSHD region gene 1 pseudogene	-2.60	0.0195
TFCP2L1	transcription factor CP2-like 1	-2.56	0.0862
RRM2	ribonucleotide reductase M2	-2.55	0.0177
EVI2A	ecotropic viral integration site 2A	-2.51	0.0926
TYMS	thymidylate synthetase	-2.51	0.0323
EAF2	ELL associated factor 2	-2.47	0.0937
ZNF367	zinc finger protein 367	-2.46	0.0018
MCM4	minichromosome maintenance complex component 4	-2.43	0.0083
MCM10	minichromosome maintenance complex component 10	-2.43	0.0527
KLF5	Kruppel-like factor 5 (intestinal)	-2.41	0.0665
CENPK	centromere protein K	-2.40	0.0656
HELLS	helicase, lymphoid-specific	-2.39	0.0190
CDC6	cell division cycle 6 homolog (S. cerevisiae)	-2.37	0.0798
TYMS	thymidylate synthetase	-2.30	0.0178
GZMH	granzyme H (cathepsin G-like 2, protein h-CCPX)	-2.29	0.0473
DDX17	DEAD (Asp-Glu-Ala-Asp) box polypeptide 17	-2.28	0.0648
MRPL43	mitochondrial ribosomal protein L43	-2.26	0.0089
HAMP	hepcidin antimicrobial peptide	-2.26	0.0816
MCM2	minichromosome maintenance complex component 2	-2.25	0.0099
GAPT	GRB2-binding adaptor protein, transmembrane	-2.24	0.0757
RRAGD	Ras-related GTP binding D	-2.22	0.0683
TREM2	triggering receptor expressed on myeloid cells 2	-2.20	0.0921
PBK	PDZ binding kinase	-2.18	0.0865
SVEP1	sushi, von Willebrand factor type A, EGF and pentraxin domain containing 1	-2.17	0.0733
TGFBR3	transforming growth factor, beta receptor III	-2.16	0.0155
PPAP2A	phosphatidic acid phosphatase type 2A	-2.15	0.0684
UHRF1	ubiquitin-like with PHD and ring finger domains 1	-2.14	0.0738
TFEC	transcription factor EC	-2.14	0.0551
PLEKHG1	pleckstrin homology domain containing, family G (with RhoGef domain) member 1	-2.14	0.0874
ZWILCH	Zwilch, kinetochore associated, homolog (Drosophila)	-2.13	0.0400
SPA17	sperm autoantigenic protein 17	-2.12	0.0464
STK17B	serine/threonine kinase 17b	-2.12	0.0461
FCGR1A	Fc fragment of IgG, high affinity Ia, receptor (CD64)	-2.12	0.0324
MAFIP	MAFF interacting protein	-2.10	0.0154
BUB1B	budding uninhibited by benzimidazoles 1 homolog beta (yeast)	-2.10	0.0420
RRM2	ribonucleotide reductase M2	-2.10	0.0415
BHLHE41	basic helix-loop-helix family, member e41	-2.07	0.0126
MCM5	minichromosome maintenance complex component 5	-2.06	0.0305
ATP8B1	ATPase, aminophospholipid transporter, class I, type 8B, member 1	-2.05	0.0653
UBE2T	ubiquitin-conjugating enzyme E2T (putative)	-2.05	0.0358
ATAD2	ATPase family, AAA domain containing 2	-2.03	0.0980
OXR1	oxidation resistance 1	-2.02	0.0456
EZR	ezrin	-2.00	0.0416
PPAP2A	phosphatidic acid phosphatase type 2A	2.04	0.0741
AQP3	aquaporin 3 (Gill blood group)	2.06	0.0844
MAD2L1	MAD2 mitotic arrest deficient-like 1 (yeast)	2.08	0.0582
GIN1	gypsy retrotransposon integrase 1	2.11	0.0373
RGS17	regulator of G-protein signaling 17	2.12	0.0074
LOC642236	similar to FRG1 protein (FSHD region gene 1 protein)	2.13	0.0203
ITGB3BP	integrin beta 3 binding protein (beta3-endonexin)	2.22	0.0323
POPDC3	popeye domain containing 3	2.28	0.0289
FCGR1B	Fc fragment of IgG, high affinity Ib, receptor (CD64)	2.28	0.0350
LOC253039	hypothetical LOC253039	2.51	0.0832
PRIM1	primase, DNA, polypeptide 1 (49kDa)	2.55	0.0039
CLDN10	claudin 10	3.30	0.0988

**Figure 4:**
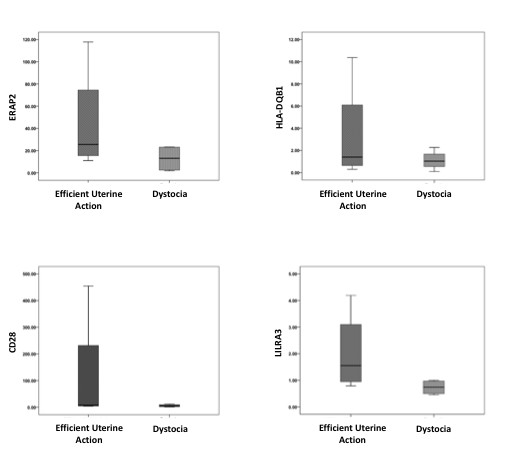
**PCR validation**. rt-PCR based validation of 4 immune related genes

As the majority of dystocia-related genes were down-regulated, these genes were examined in detail. A significant proportion of genes in this group were associated with inflammation and immune response. To validate our findings, qRT-PCR was performed on four of the immune response genes: ERAP2 and HLA-DQB1, both of which play key roles in antigen presentation; CD28, a key regulator of the TH2 T cell response and LILRA3, a regulator of monocyte and B cell activation. qRT-PCR confirmed that all four genes were down-regulated in dystocia compared to normally progressing labor (Figure 4).

## Discussion

Although dystocia represents one of the major indications for primary CS, to our knowledge, genome-wide analysis has not been applied to the study of the molecular mechanisms underlying dystocia in term nulliparous labor. High-throughput screening technologies, such as DNA microarrays have been used to improve comprehension of the major structural and metabolic transformations, which affect the myometrium from the very beginning of pregnancy until the onset of labor [18-22]. Changes in the structural and contractile genes associated with the actin cytoskeleton, focal adhesion molecules, adherens and tight junctions represent a large subset of genes that are over-expressed in pregnant, compared to non-pregnant, human myometrium [18]. Additionally, in an attempt to further decipher the causes of pre-term labor, a number of investigators have used transcriptomic approaches to examine the transition from uterine quiescence to the onset of contractions in small numbers of patients [18-22]. These studies identified a number of genes, which may be useful in predicting pre-term labor; however, none have specifically examined dystocia. Our findings demonstrate significant differences in the myometrial transcriptomic profiles of dystocic as compared to normally-progressing labors. In particular, we have demonstrated that a number of key regulators of the host immune response are down-regulated in dystocia.

The strengths of this study are mainly based around the study design whereby the inclusion criteria were strict. Patients included in this study were nulliparas in spontaneous labor. All women in the dystocia group received 30mU/min oxytocin for at least 4 hours. In addition the study was performed in a unit where the diagnosis and management of spontaneous nulliparous labor has been standardized for the last four decades [14]. A major weakness of this study is the small number of samples studied. In addition, sampling the lower segment alone may give a true global description of the dystocic transcriptome. Transcriptomic profiling has shown different myometrial gene expression patterns in lower segment compared to fundal biopsies in labour [22,23], however the majority of these studies appear to have been performed in multiparous patients and thus these finds may not be applicable to our study. Dystocia, due to ineffective myometrial contractility is much more common in women undergoing induction of labor, although induced labors were specifically excluded from this study in an attempt to compare two homogenous groups. Also the indication for induction is important as a woman undergoing labor induction following a prolonged interval of ruptured membranes is likely to have a different myometrial profile to a patient being induced for pre-eclampsia. The absence of an independent validation cohort represents another weakness of this study, although internal validation using rt-PCR was performed to address this issue.

This study further strengthens the potential argument for an underlying genetic predisposition for dystocia [6,24]. The finding that the majority of differentially expressed genes were down-regulated suggests that many of these may harbour loss-of-function mutations. The association of dystocia with an impaired immune response is also in agreement with a larger recent study by Mittal *et al *[25], who demonstrated increased expression of a number of inflammatory genes in patients with arrest of descent in the second stage, a cohort similar to the efficient uterine action group in our study. Comparison of our findings to those of Mittal *et al *[25] should be tempered by the fact that their study did not stratify for parity or mode of onset of labor. The importance of the inflammatory response in spontaneous labor cannot be overstated, since Unal *et al *[26] recently demonstrated that maternal inflammatory markers increase before the onset of spontaneous labor, suggesting that any future studies of dystocia should be stratified according to labor onset (spontaneous versus induced). Similarly, dystocia in a multiparous woman is much more likely to be secondary to obstruction than inefficient uterine action and thus likely to have a different underlying molecular profile, emphasizing the need for stratification by parity.

The identification of ERAP2 as a gene that was significantly down-regulated in dystocia is of particular interest because ERAP2 has been identified as a genetic susceptibility locus for preeclampsia in a number of different populations [27,28]. Although ERAP2 is considered to be a member of the oxytocinase subfamily of M1 aminopeptidases, it has no hydrolytic activity towards oxytocin [29]. ERAP2 which is regulated by interferon gamma appears to play a key role in the innate immune response whereby it trims various N-terminal extended precursors to major histocompatibility complex class I-presented antigenic peptides [29]. Although a number of missense single nucleotide polymorphisms (SNP) in ERAP2 have been associated with an increased risk of preeclampsia [27,28], the functional importance of these SNPs is still not fully understood. A recent study did however demonstrate that specific ERAP2 haplotypes are associated with lower levels of MHC class I expressed on the surface of B cells, suggesting that naturally occurring ERAP2 deficiency affects MHC presentation and immune response [30].

Although historical studies have suggested that labor may be easier to induce in preeclamptic patients [31,32], a number of recent reports have suggested that women with preeclampsia undergoing labor induction have higher cesarean delivery rates compared with non-preeclamptics, independent of parity or gestational age [33-35]. In a large retrospective cohort study, Kim *et al *demonstrated a significantly higher term nulliparous CS rate in induced preeclamptic patients compared to healthy controls [33]. While our study was conducted on nulliparas in spontaneous term labour these findings raise intriguing questions about the role of ERAP2 and efficient uterine action in preeclamptic patients, which warrant further investigation.

## Conclusion

Our study demonstrates an obvious difference in the myometrial transcriptomic profiles in women with dystocia and efficient uterine action but it also raises a number of questions. It is currently not possible to predict which women will develop dystocia before the onset of spontaneous labor. Due to the invasive nature of a biopsy, it would not be possible to reduce a myometrial profile to clinical utility, but it is worth noting that two of the genes significantly down-regulated in dystocic patients, ERAP2 and LILRA3, code for secreted proteins which could potentially be detected in human plasma. LILRA3 has previously been detected in rheumatoid arthritis [36]. Nevertheless, the advent of whole genome sequencing offers a platform to further investigate dystocia and to identify the genetic determinants of this complex condition, which is central to ever-increasing international CS rates and thus represents a major current public health issue.

## Competing interests

The authors declare that they have no competing interests.

## Authors' contributions

DJB conceived the study, collected the samples, performed statistical analysis and drafted the manuscript, SMG processed the samples and performed RT-PCR, ER performed statistical analysis, DOC performed RT-PCR, MR conceived the study, provided samples and drafted the manuscript, COH conceived the study, provided the samples and drafted the manuscript. All authors read and approved the final manuscript.

## Authors information

DJB, MR and COH are obstetricians and gynaecologists. DJB, SMG, ER and DOC are research scientists with an interest in biomarker identification and validation

## Funding

The study was funded by the National Maternity Hospital Research Fund

## Pre-publication history

The pre-publication history for this paper can be accessed here:

http://www.biomedcentral.com/1471-2393/11/74/prepub

## References

[B1] Brennan DJ Murphy M Robson MS O'Herlihy C The singleton, cephalic, nulliparous woman after 36 weeks of gestation: contribution to overall cesarean delivery rates Obstet Gynecol 2011 117 2 Pt 1 273 279 21252739 10.1097/AOG.0b013e318204521a

[B2] Brennan DJ Robson MS Murphy M O'Herlihy C Comparative analysis of international cesarean delivery rates using 10-group classification identifies significant variation in spontaneous labor Am J Obstet Gynecol 2009 201 3 308 e301 308 19733283 10.1016/j.ajog.2009.06.021

[B3] Lee YM Wylie BJ Simpson LL D'Alton ME Twin chorionicity and the risk of stillbirth Obstet Gynecol 2008 111 2 Pt 1 301 308 18238966 10.1097/AOG.0b013e318160d65d

[B4] Hannah ME Hannah WJ Hewson SA Hodnett ED Saigal S Willan AR Planned caesarean section versus planned vaginal birth for breech presentation at term: a randomised multicentre trial. Term Breech Trial Collaborative Group Lancet 2000 356 9239 1375 1383 10.1016/S0140-6736(00)02840-3 11052579

[B5] Riskin A Riskin-Mashiah S Bader D Kugelman A Lerner-Geva L Boyko V Reichman B Delivery mode and severe intraventricular hemorrhage in single, very low birth weight, vertex infants Obstet Gynecol 2008 112 1 21 28 10.1097/AOG.0b013e31817cfdf1 18591303

[B6] Algovik M Nilsson E Cnattingius S Lichtenstein P Nordenskjold A Westgren M Genetic influence on dystocia Acta Obstet Gynecol Scand 2004 83 9 832 837 15315594 10.1111/j.0001-6349.2004.00544.x

[B7] Cahill DJ Boylan PC O'Herlihy C Does oxytocin augmentation increase perinatal risk in primigravid labor? Am J Obstet Gynecol 1992 166 3 847 850 1550151 10.1016/0002-9378(92)91346-c

[B8] O'Driscoll K Jackson RJ Gallagher JT Prevention of prolonged labour Br Med J 1969 2 5655 477 480 10.1136/bmj.2.5655.477 5771578 PMC1983378

[B9] Lynch CM Sexton DJ Hession M Morrison JJ Obesity and mode of delivery in primigravid and multigravid women Am J Perinatol 2008 25 3 163 167 10.1055/s-2008-1061496 18300188

[B10] Treacy A Robson M O'Herlihy C Dystocia increases with advancing maternal age American journal of obstetrics and gynecology 2006 195 3 760 763 10.1016/j.ajog.2006.05.052 16949410

[B11] Sugimoto Y Yamasaki A Segi E Tsuboi K Aze Y Nishimura T Oida H Yoshida N Tanaka T Katsuyama M Failure of parturition in mice lacking the prostaglandin F receptor Science 1997 277 5326 681 683 10.1126/science.277.5326.681 9235889

[B12] Mahendroo MS Cala KM Landrum DP Russell DW Fetal death in mice lacking 5alpha-reductase type 1 caused by estrogen excess Molecular endocrinology 1997 11 7 Baltimore, Md 917 927 10.1210/me.11.7.917 9178751

[B13] Algovik M Lagercrantz J Westgren M Nordenskjold A No mutations found in candidate genes for dystocia Human reproduction 1999 14 10 Oxford, England 2451 2454 10.1093/humrep/14.10.2451 10527967

[B14] O'Driscoll K Meagher D Robson M The active management of labor 2004 New York: Mosby Year Book Limited

[B15] Team RDC A language and environment for statistical computing R Foundation for Statistical Computing 2008

[B16] Gautier L Cope L Bolstad BM Irizarry RA affy--analysis of Affymetrix GeneChip data at the probe level Bioinformatics 2004 20 3 307 315 10.1093/bioinformatics/btg405 14960456

[B17] Dolle L Adriaenssens E El Yazidi-Belkoura I Le Bourhis X Nurcombe V Hondermarck H Nerve growth factor receptors and signaling in breast cancer Curr Cancer Drug Targets 2004 4 6 463 470 10.2174/1568009043332853 15379632

[B18] Breuiller-Fouche M Germain G Gene and protein expression in the myometrium in pregnancy and labor Reproduction 2006 131 5 Cambridge, England 837 850 10.1530/rep.1.00725 16672349

[B19] Esplin MS Fausett MB Peltier MR Hamblin S Silver RM Branch DW Adashi EY Whiting D The use of cDNA microarray to identify differentially expressed labor-associated genes within the human myometrium during labor American journal of obstetrics and gynecology 2005 193 2 404 413 10.1016/j.ajog.2004.12.021 16098862

[B20] Havelock JC Keller P Muleba N Mayhew BA Casey BM Rainey WE Word RA Human myometrial gene expression before and during parturition Biology of reproduction 2005 72 3 707 719 10.1095/biolreprod.104.032979 15509731

[B21] Charpigny G Leroy MJ Breuiller-Fouche M Tanfin Z Mhaouty-Kodja S Robin P Leiber D Cohen-Tannoudji J Cabrol D Barberis C A functional genomic study to identify differential gene expression in the preterm and term human myometrium Biology of reproduction 2003 68 6 2289 2296 12606369 10.1095/biolreprod.102.013763

[B22] Bukowski R Hankins GD Saade GR Anderson GD Thornton S Labor-associated gene expression in the human uterine fundus, lower segment, and cervix PLoS medicine 2006 3 6 e169 10.1371/journal.pmed.0030169 16768543 PMC1475650

[B23] Havelock J Keller P Muleba N Mayhew B Casey B Rainey W Word R Human myometrial gene expression before and during parturition Biology of reproduction 2005 72 3 707 719 10.1095/biolreprod.104.032979 15509731

[B24] Berg-Lekås ML Högberg U Winkvist A Familial occurrence of dystocia Am J Obstet Gynecol 1998 179 1 117 121 10.1016/S0002-9378(98)70260-1 9704775

[B25] Mittal P Romero R Tarca AL Draghici S Nhan-Chang CL Chaiworapongsa T Hotra J Gomez R Kusanovic JP Lee DC A molecular signature of an arrest of descent in human parturition Am J Obstet Gynecol 2011 204 2 177 e115 133 21284969 10.1016/j.ajog.2010.09.025 PMC3053040

[B26] Unal ER Cierny JT Roedner C Newman R Goetzl L Maternal inflammation in spontaneous term labor Am J Obstet Gynecol 2011 204 3 223 e221 225 21376162 10.1016/j.ajog.2011.01.002

[B27] Johnson MP Roten LT Dyer TD East CE Forsmo S Blangero J Brennecke SP Austgulen R Moses EK The ERAP2 gene is associated with preeclampsia in Australian and Norwegian populations Hum Genet 2009 126 5 655 666 10.1007/s00439-009-0714-x 19578876 PMC2783187

[B28] Hill LD Hilliard DD York TP Srinivas S Kusanovic JP Gomez R Elovitz MA Romero R Strauss JF Fetal ERAP2 variation is associated with preeclampsia in African Americans in a case-control study BMC Med Genet 2011 12 1 64 21569342 10.1186/1471-2350-12-64 PMC3103419

[B29] Tanioka T Hattori A Masuda S Nomura Y Nakayama H Mizutani S Tsujimoto M Human leukocyte-derived arginine aminopeptidase. The third member of the oxytocinase subfamily of aminopeptidases J Biol Chem 2003 278 34 32275 32283 10.1074/jbc.M305076200 12799365

[B30] Andres AM Dennis MY Kretzschmar WW Cannons JL Lee-Lin SQ Hurle B Schwartzberg PL Williamson SH Bustamante CD Nielsen R Balancing selection maintains a form of ERAP2 that undergoes nonsense-mediated decay and affects antigen presentation PLoS Genet 2010 6 10 e1001157 10.1371/journal.pgen.1001157 20976248 PMC2954825

[B31] Taylor ES Bruns PD Anker RM Drose VE Correlation of urinary estrogen-pregnanediol excretion with uterine motility during pregnancy Am J Obstet Gynecol 1955 70 4 894 909 13258678 10.1016/s0002-9378(16)37842-5

[B32] Zuspan FP Talledo E Factors affecting delivery in eclampsia: the condition of the cervix and uterine activity Am J Obstet Gynecol 1968 100 5 672 685 5638487 10.1016/s0002-9378(15)33392-5

[B33] Kim LH Cheng YW Delaney S Jelin AC Caughey AB Is preeclampsia associated with an increased risk of cesarean delivery if labor is induced? J Matern Fetal Neonatal Med 2010 23 5 383 388 10.3109/14767050903168432 19951010

[B34] Ben-Haroush A Yogev Y Glickman H Kaplan B Hod M Bar J Mode of delivery in pregnant women with hypertensive disorders and unfavorable cervix following induction of labor with vaginal application of prostaglandin E Acta Obstet Gynecol Scand 2005 84 7 665 671 15954877 10.1111/j.0001-6349.2005.00681.x

[B35] Xenakis EM Piper JM Field N Conway D Langer O Preeclampsia: is induction of labor more successful? Obstetrics and gynecology 1997 89 4 600 603 10.1016/S0029-7844(97)00043-4 9083320

[B36] An H Chandra V Piraino B Borges L Geczy C McNeil HP Bryant K Tedla N Soluble LILRA3, a potential natural antiinflammatory protein, is increased in patients with rheumatoid arthritis and is tightly regulated by interleukin 10, tumor necrosis factor-alpha, and interferon-gamma J Rheumatol 2010 37 8 1596 1606 10.3899/jrheum.091119 20595277

